# The risk for celiac disease after Covid-19 infection

**DOI:** 10.1186/s12876-023-02795-3

**Published:** 2023-05-22

**Authors:** Jesper Lexner, Ylva Lindroth, Klas Sjöberg

**Affiliations:** 1 Department of Gastroenterology and Nutrition, Department of Clinical Sciences, Skåne University Hospital, Lund University, Malmö, Sweden; 2 Division of Medical Microbiology, Department of Laboratory Medicine, Skåne University Hospital, Lund University, Lund, Sweden

**Keywords:** Celiac disease, Covid-19, Incidence, Respiratory infections, Risk

## Abstract

**Background:**

Celiac disease (CD) is an autoimmune disease leading to gastrointestinal symptoms and mineral deficiencies. The pathogenetic mechanisms, besides the clear HLA association, are elusive. Among environmental factors infections have been proposed. Covid-19 infection results in a systemic inflammatory response that often also involves the gastrointestinal tract. The aim of the present study was to investigate whether Covid-19 infection could increase the risk for CD.

**Patients and methods:**

All patients, both children and adults, in the county Skåne (1.4 million citizens) in southern Sweden with newly diagnosed biopsy- or serology-verified CD or a positive tissue transglutaminase antibody test (tTG-ab) during 2016–2021 were identified from registries at the Departments of Pathology and Immunology, respectively. Patients with a positive Covid-19 PCR or antigen test in 2020 and 2021 were identified from the Public Health Agency of Sweden.

**Results:**

During the Covid-19 pandemic (March 2020 – December 2021), there were 201 050 cases of Covid-19 and 568 patients with biopsy- or serology-verified CD or a first-time positive tTG-ab tests, of which 35 patients had been infected with Covid-19 before CD. The incidence of verified CD and tTG-ab positivity was lower in comparison to before the pandemic (May 2018 – February 2020; 22.5 vs. 25.5 cases per 100 000 person-years, respectively, incidence rate difference (IRD) -3.0, 95% CI -5.7 – -0.3, p = 0.028). The incidence of verified CD and tTG-ab positivity in patients with and without prior Covid-19 infection was 21.1 and 22.4 cases per 100 000 person-years, respectively (IRD − 1.3, 95% CI -8.5–5.9, p = 0.75).

**Conclusions:**

Our results indicate that Covid-19 is not a risk factor for CD development. While gastrointestinal infections seem to be an important part of the CD pathogenesis, respiratory infections probably are of less relevance.

## Introduction

Celiac disease (CD) is a chronic autoimmune disorder triggered by the consumption of gluten in genetically predisposed individuals [1]. The incidence of CD has increased substantially during the 1900s and the beginning of the 2000s [2, 3]. About 1% of the population worldwide is affected by the disease, but it is more common in Scandinavia [4]. A screening study of Swedish teenagers revealed a prevalence of 2–3% [5].

Dietary gluten consumption and genetical predisposition in the form of the HLA-DQ2 or -DQ8 alleles are seen in almost all CD cases. It is estimated that around 50% of the Swedish population carry the HLA-DQ2 or -DQ8 alleles [6], but only 2–3% of the population is affected by CD [5]. Neither can genetic factors alone explain the rapidly increasing incidence. Hence, environmental factors constitute an important part in the CD pathogenesis [7]. Among these are dietary factors. A high gluten consumption in early childhood has been linked to increased risk of CD [8]. Other relevant environmental factors seem to be the intestinal microbiota and viral infections [1].

There is growing evidence that infections early in life increase the risk of CD, as shown in both retrospective and prospective observational studies [9–12]. The increased risk has generally been ascribed to gastrointestinal infections [9], and the TEDDY Study Group has shown that a gastrointestinal infection in early childhood increases the risk of CD in the proceeding three months [13]. Frequent rotavirus infections were associated with CD autoimmunity in a U.S. cohort [14]. As of lately, prospective Scandinavian studies on genetically predisposed children have shown higher frequencies of enterovirus infections before CD onset in children [15–17]. Less is known whether other forms of infections can trigger CD onset. A few studies have found an increased risk of CD after respiratory infections in young children [18, 19], whereas others only reported a very slightly increased risk [10] or no risk at all [13]. In a Swedish study, children with CD were more likely to have been treated at the hospital for respiratory syncytial virus (RSV) before the CD diagnosis [20]. Of interest, a Norwegian study of people of all ages reported a higher incidence of CD following both seasonal and pandemic influenza infection [21].

Coronavirus disease-19 (Covid-19) was declared a pandemic by WHO in March 2020. As of 31 Jan 2023, there has been over 753 million cases of Covid-19 worldwide [22]. The virus may cause a systemic inflammatory response, sometimes with severe respiratory symptoms and even death. Severe acute respiratory syndrome-like coronavirus 2 (SARS-CoV-2) can be found in the feces of infected patients. About 10% of infected patients have been estimated to suffer from diarrhea, although the frequency of this symptom ranges from 2 to 50% in different studies [23]. Since there have been cases of Guillain-Barré syndrome after Covid-19 infection and reports that some patients produce autoantibodies during the infection concerns have been raised that Covid-19 might trigger autoimmune diseases [24, 25]. A recent meta-analysis reported increased risk of diabetes after Covid-19 infection. This could be seen both in diabetes mellitus type 1 and 2 [26]. Diabetes mellitus type 1 and CD have several common pathogenetic mechanisms, both genetic and environmental [7], but it is not known if Covid-19 can trigger CD. Covid-19 promotes a cytokine storm which may result in intestinal epithelial damage that theoretically is suggested to contribute to the onset of CD [27].

During a pandemic many individuals are infected almost simultaneously and with varying disease courses. The Covid-19 pandemic gives a unique opportunity to study whether a new virus that has never infected humanity before might have an impact on the onset of an immune driven disease. Consequently, we aimed to investigate whether Covid-19 infection could increase the risk of CD.

## Patients and methods

A retrospective observational cohort study comprising both children and adults was designed to investigate if Covid-19 infection affects the risk of subsequent CD. All cases of CD between 2016 and 2021 and Covid-19 between 2020 and 2021 in Region Skåne, a county in southern Sweden, were identified. There were 1.32 million residents in the region in 2016 and 1.40 million in 2021 [28]. All residents in Sweden have their own unique personal identity number (PIN), given at birth and to immigrants to the country. The number corresponds to date of birth and gender and can be used to identify individuals within the healthcare registries. The occurrence of CD and Covid-19 could be connected to the right individuals in this way. To scrutinize whether Covid-19 could trigger CD, patients who had their disease onset after the Covid-19 diagnosis were compared to CD patients without prior infection. The first verified case of Covid-19 in Region Skåne on 6 March 2020, marked the start of the study and the inclusion of new cases was stopped on 31 December 2021. For comparison, the yearly incidence of CD from 2016 to 2021 was examined to identify possible trends in CD incidence prior to the pandemic. Furthermore, the monthly incidence of CD during the Covid-19 pandemic was estimated and compared with the corresponding time period before the Covid-19 pandemic, from May 2018 to February 2020.

Biopsy-verified CD cases were retrieved from the registries at the pathology departments in Region Skåne. To identify CD cases, Systematized Nomenclature of Medicine (SNOMED) clinical terms D6218 (celiac disease), M58005 (partial villous atrophy) and M58006 (subtotal/total villous atrophy) were used. M58007 (total villous atrophy) is not used in Region Skåne. The SNOMED topography codes in the database search were T64 (duodenum) and T65 (jejunum/ileum). Due to variations in time interval between the arrival and examination of the specimen, the biopsy’s arrival date to the pathology department (usually the day after the gastroscopy) was used as the date of CD diagnosis. To exclude control biopsies during follow-up all biopsies fulfilling the above criteria between 2010 and 2021 were identified and the date of the first biopsy indicating CD was used as the date of diagnosis. The use of villous atrophy in biopsy reports from Swedish pathology departments has been validated previously, with a specificity of 95% for a clinical CD diagnosis [29].

Furthermore, patients with a positive IgA or IgG tissue transglutaminase antibody (tTG-ab) serology were identified. tTG-ab titers were analyzed at the Department of Clinical immunology at Skåne University Hospital according to established procedures. Levels of tTG-ab > 10 kilo-arbitrary units per liter (kU/L) were considered positive. Patients with a positive tTG-ab serology before the start of the study were excluded.

A verified CD diagnosis was based on either a biopsy with villous atrophy or a serology-based diagnosis, defined as two separate tTG-ab tests more than 10 times above the upper limit of normal. This is in accordance with the European Society for Pediatric Gastroenterology, Hepatology, and Nutrition (ESPGHAN) criteria from 2012 for serology-based diagnosis in children [30] and from 2020 this strategy is also recommended in adults in Sweden according to new revised guidelines [31]. In cases with both a biopsy and serology indicating CD, we used the first positive test as the date of onset of CD.

Separately, an additional analysis during the period 2016-21 was performed in which patients with a first-time positive tTG-ab test was included regardless of biopsy status. To be able to identify both onset of manifest CD or any increase in tTG-ab-status a combined outcome was used, i.e., presence of either a biopsy indicating CD or a first positive tTG-ab (above the reference cut-off 10 kU/L).

It was mandatory for all healthcare providers, both public and private, to report all patients that tested positive for Covid-19 during the pandemic to the Public Health Agency of Sweden. The Covid-19 cases in Region Skåne from 2020 to 2021 were retrieved from this registry. If a patient had several positive tests, the first positive test was used as the date of Covid-19 diagnosis for our analyses. SARS-CoV-2 RNA was detected using polymerase chain reaction (PCR) or antigen tests. The PCR was based on the method developed by Corman et al. [32].

Incidence calculations were carried out based on monthly population data from Statistics Sweden [28]. Comparisons were made before and during the pandemic, and between patients with CD after Covid-19 versus CD without previous Covid-19. Incidence rate differences were calculated with Stata´s *ir* command and tested using Chi2. Groupwise comparisons for baseline characteristics were done with Chi2 for binary data and Student’s T-test for continuous outcomes that were normally distributed. Normal distribution was checked graphically and with calculation of skewness. Statistical analyses were performed with Stata Statistical Software 17.0 for Windows (StataCorp, College Station, Texas). A p-value < 0.05 was considered significant in all analyses.

The study was approved by the Swedish Ethical Review Authority (protocol number 2021–04648). Since the present study was strictly register-based informed consent was not required. Waiver of informed consent was approved by the Swedish Ethical Review Authority. All methods used in this study were carried out in accordance with relevant guidelines and regulations.

## Results

**Fig. 1:**
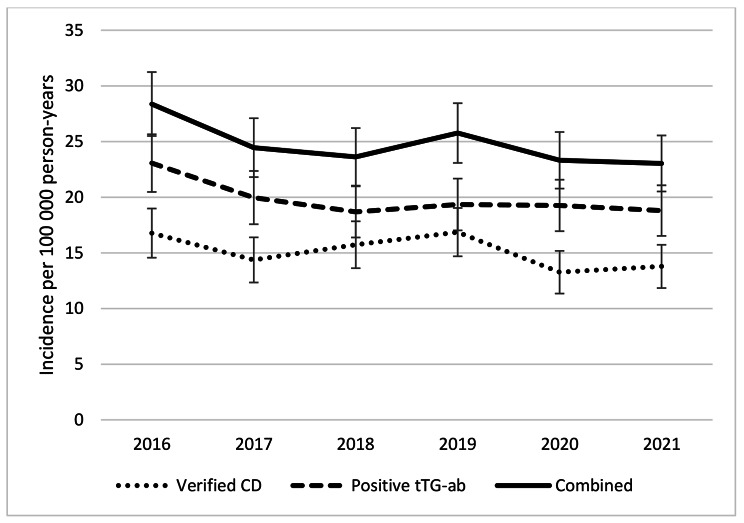
The yearly incidence of biopsy- and serology-verified celiac disease (CD) decreased from 2016 to 2021 (p = 0.046), as did the frequency of first-time positive tissue transglutaminase antibody (tTG-ab) tests (p = 0.015) and the number of cases with the combined outcome measure of either verified CD or positive tTG-ab (p = 0.006). Bars indicate 95% confidence intervals

To get an overview of the CD epidemiology in the region, the yearly incidence of the disease from 2016 to 2021 was determined. There were 222 cases of biopsy- or serology-verified CD in 2016 and 193 cases in 2021. The incidence declined during the period, from 16.8 in 2016 to 13.8 cases per 100 000 person-years in 2021 (incidence rate difference (IRD) -3.0, 95% confidence interval (CI) -5.9 – -0.5, p = 0.046; Fig. 1). A similar decrease was observed in the number of patients with a first-time positive tTG-ab test, which went from 305 cases in 2016 to 263 cases in 2021 (23.1 and 18.8 cases per 100 000 person-years, respectively, IRD − 4.3, 95% CI -7.7 – -0.8, p = 0.015). Notably, the number of performed tTG-ab tests decreased during this period, from 26 576 tests in 2016 to 22 765 in 2021. The proportion of positive tests remained unchanged (1.15% in 2016, 1.16% in 2021, p = 0.94). Both verified CD and the number of patients with positive tTG-ab had similar incidence patterns over time. There were 375 combined cases in 2016 compared to 322 in 2021 (28.4 and 23.0 cases per 100 000 person-years, respectively, IRD − 5.3, 95% CI -9.2 – -1.5, p = 0.006). In the upcoming analyses the combined outcome is reported.

The monthly incidence of the combined outcome (verified CD or tTG-ab positivity) during the Covid-19 pandemic was determined, from March 2020 to December 2021, and compared with the same time before the pandemic (May 2018 to February 2020). In total, there were 639 cases before and 573 cases during the pandemic. Of the patients, 62% and 58% were women before and during the pandemic, respectively (p = 0.085). They had a mean age of 25.7 years (range 0.9–93.7 years) and 24.6 years (range 0.8–87.8 years), respectively (mean difference − 1.1 years, 95% CI -3.6–1.4, p = 0.39). The incidence decreased from 25.5 cases per 100 000 person-years before the pandemic to 22.5 during the pandemic (IRD − 3.0 cases per 100 000 person-years, 95% CI -5.7 – -0.3, p = 0.028; Table 1). Stratification on age revealed that the incidence decreased with 3.5 cases per 100 000 person-years among adults (p = 0.003) and with 1.4 cases per 100 000 person-years among children, although the latter was non-significant (p = 0.77). There was also a decreased incidence among women (IRD − 5.9, p = 0.005) and a small non-significant decrease among men (IRD − 0.10, p = 0.95; Table 1).

**Table 1:** Incidence of verified celiac disease or a positive tissue transglutaminase antibody test before the Covid-19 pandemic (May 2018 – February 2020) and during the pandemic (March 2020 – December 2021)

	Incidence before pandemic	Incidence during pandemic	Incidence rate difference (95% CI)	p-value
Total	25.5	22.5	-3.0 (-5.7 – -0.3)	**0.028**
> 18 years	15.6	12.1	-3.5 (-5.8 – -1.2)	**0.003**
< 18 years	62.6	61.2	-1.4 (-10.8–8.1)	0.77
Women	31.8	25.9	-5.9 (-10.1 – -1.8)	**0.005**
Men	19.2	19.1	-0.1 (-3.5–3.3)	0.95

Incidence rates as cases per 100 000 person-years

**Fig. 2:**
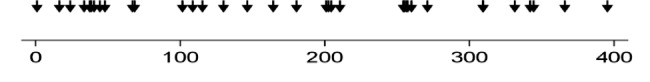
Days from Covid-19 to verified celiac disease diagnosis or first-time positive tissue transglutaminase antibody test (n = 35)

In the population of 1.4 million citizens in the region, 201 050 people were diagnosed with Covid-19 from the start of the pandemic until the end of the study on 31 December 2021. They had a mean age of 38.9 years (range 1 day – 108 years old) and 51% were women. There were 568 cases of verified CD or tTG-ab positivity during this time. Of these, 35 patients were infected with Covid-19 before CD or tTG-ab positivity. The incidence in patients with previous Covid-19 infection was 21.1 cases per 100 000 person-years. Among patients who got CD or tTG-ab positivity without previously being diagnosed with Covid-19, the incidence was 22.4 cases per 100 000 person-years. There was no difference between the two groups (IRD − 1.3 cases per 100 000 person-years, 95% CI -8.5–5.9, p = 0.75; Table 2). The time between Covid-19 infection and CD or tTG-ab positivity was evenly distributed (Fig. 2) with half of the patients being identified during the first six months after the Covid-19 diagnosis, and the rest during the upcoming six months (except for one patient diagnosed after 395 days).

**Table 2:** Incidence of verified celiac disease or a positive tissue transglutaminase antibody test (CD) and baseline characteristics among patients without a previous Covid-19 infection and with a previous infection

	CD without previous Covid-19	CD with previous Covid-19	Difference (95% CI)**	p-value
Cases (no)	533	35		
Person-years (no)	2 383 813	166 098		
Incidence rate*	22.4	21.1	-1.3 (-8.5–5.9)	0.75
Gender (% women)	57.4%	57.1%		0.98
Mean age (range)	23.9 years (0.8–87.8)	32.9 years (7.0–86.0)	9.0 years (1.3–16.7)	**0.022**

* Incidence rate as cases per 100 000 person-years. ** Incidence rate difference and mean difference in age with 95% confidence intervals

The group who developed verified CD or tTG-ab positivity after Covid-19 infection did not differ in gender compared to those without a previous Covid-19 infection. There were 57% females in both groups (Table 2). Those who got CD or tTG-ab positivity after Covid-19 were older; they had a mean age of 32.9 years compared to 23.9 years (mean difference 9.0 years, 95% CI 1.3–16.7, p = 0.022). Since this subgroup consisted of only 35 patients, stratification on age was not possible. In this cohort 19 patients were adults (54%). Seventeen had verified CD (49%) and the rest a positive tTG-ab test. Of the patients with verified CD eight had a biopsy-verified diagnosis and nine were based on serology (two tests at least 10 times the upper limit of normal).

## Discussion

There is growing evidence that infections may constitute an important part of CD pathogenesis [9–21]. Less is known about which specific pathogens that are of relevance. The recent pandemic gave an opportunity to study this further. To the best of our knowledge, this is the first epidemiological study that has investigated if Covid-19 infection can trigger CD. We did not find a higher incidence of CD or tTG-ab positivity in patients with recent Covid-19 infection. In fact, there was a general decrease in CD incidence during the pandemic compared to before. Neither could we find any clustering in time of CD cases or tTG-ab positive patients in relation to their previous Covid-19 infection. The cases were evenly distributed over time after the infection. If Covid-19 would trigger CD, one would expect a clustering of cases at a certain time span after the infection. Altogether, our results indicate that Covid-19 is not a risk factor for CD development.

The decreased incidence of CD could be due to reduced accessibility to healthcare during the pandemic, but the fact that the decline started several years before the pandemic makes this less likely. A reduced access to invasive procedures such as gastroscopy could be expected, but we also found a decrease in positive tTG-ab cases. Since the proportion of tests with a positive outcome remained unchanged there is no indication that any increase in tTG-ab positivity did occur. The incidence of CD in Sweden started to decline already before the start of our study period [33]. Consequently, it is not probable that the observed reduction in CD cases is due to the pandemic alone but also to a shift in the incidence over a longer time period – already before the period included in the present study – that is not related to Covid-19. There is no clear indication that Covid-19 has increased the number of CD cases.

A German study by Lingel et al. [34] in adults compared tTG-ab levels in 80 Covid-19 convalescents and 39 controls three months after the Covid-19 infection. The levels were higher in Covid-19 convalescents than in controls, which contrasts with our results. This observation could indicate that at least tTG-ab positivity can develop after a Covid-19 infection. However, most patients just had slightly elevated values of unknown clinical significance and no one more than 10 times the upper limit of normal (> 100 kU/L). There was no data on tTG-ab levels before the infection and no data on clinical symptoms. The cases with Covid-19 infection also had more diabetes and autoimmune diseases in general than the controls. If the result would remain after adjustment for the differences in comorbidity between the controls and Covid-19 infected, it could represent a temporary production of tTG-ab which does not cause CD. A small Italian study has previously reported that some children with an infection produce tTG-ab that normalize after recovering from the infection despite continuing a gluten-containing diet [35]. In another Italian investigation in children from Genoa the number of new cases with CD during a two-year period before the Covid-19 pandemic was compared to the two years of the pandemic. Before the pandemic 228 patients could be identified compared to 195 during the pandemic indicating that the pandemic did not increase the risk for CD [36]. This is in line with the observation in the present study.

While several studies report that gastroenteritis in children seems to trigger CD [9, 13–17], studies on the risk after respiratory infections are fewer and contradictory. In children at high genetic risk for CD, Kemppainen et al. [13] found no association with CD, while Auricchio et al. [18] reported an increased risk for CD among children. Mårild et al. [10] reported an association in young children but with very small odds ratios. Kårhus et al. [21] have investigated the risk of CD one year or later after an influenza infection and found a slightly increased risk. According to these results it seems less likely that predominantly respiratory infections confer the same risk as gastrointestinal when it comes to the risk for CD.

We did not have any information about Covid-19 vaccination status in the study. The majority of Swedish citizens received the first vaccine dose during early summer 2021 [37], a minor part of the study period. Thurm et al. [38] found that vaccination did not cause tTG-ab seroconversion four months after the first dose. Previous studies have not found an increase in CD incidence following rotavirus vaccination [13, 39]. If vaccination would reduce a potential excess risk caused by Covid-19, one would expect an increase in CD incidence, especially with previous Covid-19 infection, during the beginning of the pandemic and later a decrease, something that was not observed in this study. Consequently, it is not very likely that our results are affected by Covid-19 vaccination.

Our results are strengthened by the rather large cohort of 1.4 million citizens. Sweden’s strategy to mitigate the Covid-19 spread and protect the elderly, instead of large-scale lockdowns, may have resulted in a rather large proportion of Covid-19 infections which theoretically could have increased the chance of finding an effect in our study [40]. By including both patients with verified CD and tTG-ab positivity the number of affected cases could be maximized, and the true timing of disease onset estimated more accurately. Since there might be a delay from symptom onset to diagnosis there is still a risk that the onset is not estimated optimally, but this would affect the Covid-19-dependent CD cases and non-Covid-19 cases to the same extent.

Remarkably, in this cohort of 1.4 million citizens, of whom more than 200 000 had been diagnosed with Covid-19, only 35 patients got verified CD or tTG-ab positivity after the Covid-19 infection. In an *a priori* power analysis it was estimated that, given a baseline incidence around 55 CD cases per 100 000 person-years [21] and an odds ratio of 1.33 for the CD incidence in infected versus non-infected [13], a cohort of 1.4 million would be sufficient. The incidence in our study was lower than that, so to detect a significant difference the odds ratio would have had to be a little higher. There was no tendency whatsoever towards an increased risk in our study, although we cannot exclude a potential small excess risk.

Most previous investigations on the association between infections and CD have studied young children and rarely adults [9–20]. Since Covid-19 infection results in a more severe disease course in adults it is logical to study the association between Covid-19 and CD in an adult population. From this respect it is intriguing that the age of onset was higher in the group with previous Covid-19. However, it could simply be a result of more frequent Covid-19 testing in adults due to more severe symptomatology and government recommendations on whom should be tested. Since the group with previous Covid-19 differed slightly in mean age compared to those without previous infection, the results get a bit more difficult to interpret. A somewhat larger incidence in the younger group with no prior Covid-19 may be expected. The age difference is one of the major limitations of the study. Any larger excess risk ought to be detectable, though. Neither can it be excluded that Covid-19 might trigger CD in some age groups but not others, although the general decline in CD incidence argues against this theory. The number of CD cases with previous Covid-19 infection was lower than expected. Hence, stratification on age or gender was unfortunately not possible.

Another potential limitation is the fact that the follow-up time could be too short. The exact timing of CD onset following an infection is unknown. Whereas Kårhus et al. [21] reported an increased risk of CD one year or later following influenza, Kemppainen et al. [13] noticed tTG-ab seroconversion already three months after a gastroenteritis in children. It might be possible that the different disease courses in gastrointestinal and respiratory infections trigger CD through separate pathogenetic mechanisms affecting the onset of disease. Alternatively, it is a consequence of prospective versus retrospective study designs. Nevertheless, our study indicates that the risk of CD onset early after a Covid-19 infection is not increased.

It is not known to which extent patients might have had Covid-19 without being tested. During the first half of 2020 testing was focused on people at increased risk of severe Covid-19 such as elderly and patients requiring hospital care, while testing was less available for the general population. Testing was also restricted due to shortage of testing equipment. During the latter half of 2020 and onwards, testing was recommended for the general population [41]. Although there have been periods when the maximum testing capacity was exceeded, it is primarily the first half of 2020 where the Covid-19 cases in our study might have been missed, making it harder to detect if infection increases the risk of CD. Still, we observed a reduced incidence of CD during the pandemic arguing against the theory that missed cases due to inadequate testing could explain the findings.

## Conclusions

Our results indicate that Covid-19 is not a risk factor for CD development. While gastrointestinal infections seem to be an important part of the CD pathogenesis, respiratory infections probably are of less relevance. Although there are some limitations, this study sheds some important light on the issue. There is a need for further research to confirm our results. Such studies should be case-controlled to account for differences in age among patients with CD and Covid-19. Still, there will be an issue of Covid-19 underdiagnosis due to varying accessibility to testing during the pandemic. Ideally, further studies would be prospective to identify more Covid-19 cases and to ensure that Covid-19 preceded CD development. Possible differences in children and adults should also be taken into consideration.

## Data Availability

The datasets generated and analyzed during the current study are not publicly available due to information that could compromise patient privacy but are available from the corresponding author on reasonable request.

## List of abbreviations

CD: Celiac disease
Covid-19: Coronavirus disease-19
SARS-CoV-2: Severe acute respiratory syndrome-like coronavirus 2
tTG-ab: tissue transglutaminase antibody
